# Water Hyacinth *Eichhornia crassipes* (Mart.) Solms-Laubach Dynamics and Succession in the Nyanza Gulf of Lake Victoria (East Africa): Implications for Water Quality and Biodiversity Conservation

**DOI:** 10.1100/2012/106429

**Published:** 2012-04-19

**Authors:** John Gichuki, Reuben Omondi, Priscillar Boera, Tom Okorut, Ally Said Matano, Tsuma Jembe, Ayub Ofulla

**Affiliations:** ^1^Kenya Marine and Fisheries Research Institute, P.O. Box 1881, Kisumu 40100, Kenya; ^2^Big Valley Rancheria Band of Pomo Indians, 2726 Mission Rancheria Road, Lake Port, CA 95453-9637, USA; ^3^Lake Victoria Basin Commission, P.O. Box 1510, Kisumu 40100, Kenya; ^4^Maseno University, P.O. Box Private Bag Maseno, Kenya

## Abstract

This study, conducted in Nyanza Gulf of Lake Victoria, assessed ecological succession and dynamic status of water hyacinth. Results show that water hyacinth is the genesis of macrophyte succession. On establishment, water hyacinth mats are first invaded by native emergent macrophytes, *Ipomoea aquatica* Forsk., and *Enydra fluctuans* Lour., during early stages of succession. This is followed by hippo grass *Vossia cuspidata* (Roxb.) Griff. in mid- and late stages whose population peaks during climax stages of succession with concomitant decrease in water hyacinth biomass. Hippo grass depends on water hyacinth for buoyancy, anchorage, and nutrients. The study concludes that macrophyte succession alters aquatic biodiversity and that, since water hyacinth infestation and attendant succession are a symptom of broader watershed management and pollution problems, aquatic macrophyte control should include reduction of nutrient loads and implementing multifaceted approach that incorporates biological agents, mechanical/manual control with utilization of harvested weed for cottage industry by local communities.

## 1. Introduction

Macrophytes are higher plants that grow in ecosystems whose formation has been dominated by water and whose processes and characteristics are largely controlled by water. Macrophytes can be subdivided into four groups on the basis of their water requirements and habitats. Submerged macrophytes are those that are completely covered with water but rooted in the substrate, for example, *Potamogeton schweinfurthii *A. Benn.

Floating leafed macrophytes are those that are rooted but have floating leaves, for example, *Nymphaea lotus *thumb. Emergent macrophytes are rooted plants with their principal photosynthetic surfaces projecting above the water, for example, *Cyperus papyrus *L. Finally, the free-floating macrophytes are those that float on the water surface, for example, water hyacinth* Eichhornia crassipes *(Mart.) Solms-Laubach and water fern/water velvet* Azolla pinnata *Decne ex Mett. Factors that influence the establishment and distribution of macrophytes include depth, topography, and type of substrate, exposure to currents and/or wind, and water turbidity [1].

Water hyacinth *Eichhornia crassipes*, a perennial aquatic herb which belongs to the pickerelweed family (Pontederiaceae), is a native of tropical America. This assumption is based on the prevalence of other species of *Eichhornia* spp. particularly the more primitive *Eichhornia paniculata* (Spreng.) Solms and *Eichhornia paradoxa *(Mart.) Solms, in this area. It has been classified as one of the worst aquatic weeds in the world [2]. The weed has spread to many parts of the world due to its beautiful large purple and violet (lavender) flowers similar to orchids that make it a popular ornamental plant for ponds [3]. In the areas bordering Lake Victoria, the weed was first recorded in Lake Kyoga (Uganda) in May 1988 [4]. Within Lake Victoria, it was observed in the Ugandan sector in 1989 ([5–8] Tanzania in 1989 [9], and Kenya in 1990 [10, 11]). In the Kagera River of Rwanda, the water hyacinth was recorded in 1991 [12] though it was believed to have been present in this area since at least the early 1980s ([13, 14]). It was present upstream of Lake Victoria on European plantations within the Kagera catchment since at least the 1940s and reached nuisance levels here, choking riverine wetland lakes, by the 1980s. Migration via the Kagera river was the most likely point of entry for water hyacinth into Lake Victoria. The weed has flourished in Lake Victoria due to absence of natural predators as insects, fish, and other biota and due to favorable environmental conditions. It is associated with major negative economic and ecological impacts to the Lake Victoria region.

The weed forms thick mats over the infested water bodies causing obstruction to economic development activities and impacting negatively on the indigenous aquatic biodiversity. Furthermore, the weed affects the conditions of the water body and life of the flora and fauna in them. Floating mats of water hyacinth for example drastically curtail the penetration of light into the aquatic ecosystem thus inhabiting the growth of phytoplankton.

Initially, efforts to control water hyacinth in Lake Victoria during the early 1990s were of limited success and were primarily directed at manually removing water hyacinth and conducting public awareness exercises. In the mid-late 1990s, management to combat water hyacinth increased with efforts such as the Lake Victoria Environmental Management Program (LVEMP) and US Agency for International Development (USAID) funding for coordination efforts by Clean Lakes, Inc. (Martinez, CA, USA). Control actions included biocontrol using *Neochetina bruchi* and *N. eichhorniae* water hyacinth weevils and mechanical control using large harvesting and chopping boats [7]. Operational water hyacinth control through the use of herbicides was not implemented in the region.

Despite water hyacinth's invasive nature and dominance in Lake Victoria in the 1990s, water hyacinth largely disappeared from Lake Victoria by the end of 1999. For instance, no water hyacinth was found on the Gulf from April 2002 until October 2004, only appearing again at the next measurement date of December 2005. Various hypotheses have been proposed on its rapid disappearance including the introduction of water hyacinth weevils [15, 16], effects of the *El Nino* weather of 1997/1998 [17], or a combination of interacting factors involving *El Nino* of 1997/1998, and biocontrol by weevils [18]. 

Here, we describe a form of macrophyte ecological succession which culminated in the control of water hyacinth in the Nyanza Gulf of Lake Victoria in 2008.

## 2. Methodology

### 2.1. Study Area

**Figure 1:**
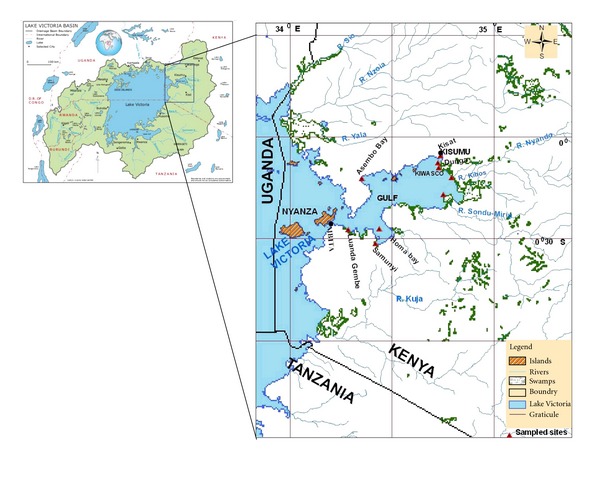
Map of the Nyanza Gulf, Lake Victoria showing the sampling sites.

The study was carried out between September and December 2008. The study sites are displayed in Figure 1. The following areas were sampled, namely, Off Kibos, Dunga beach, next to Osienala Headquarters, Sondu Miriu, Homa Bay, Oluch river mouth, Lwanda Gembe, and Asembo Bay. The study was carried out at specific areas in the Nyanza Gulf where the populations of major macrophyte species were sighted. The zones were divided into 3 zones, namely, Hippo grass *Vossia cuspidata* (Roxb.) Griff. zone, Water hyacinth *Eichhornia crassipes *(Mart.) Solms-Laubach zone, and Hippo grass/water hyacinth mixture. In most of the cases, the macrophyte mats could not be penetrated by the boat, and thus 2 zones were sampled, namely, interphase zone and open water zone. The open water zone was always located 50 metres away from the mats. Where possible, a third zone was sampled, namely, within macrophyte zone (hippo grass/water hyacinth mixture).

### 2.2. Physical and Chemical Parameters

Geographical coordinates were determined using a GPS Garmin GPS II Plus. Turbidity was measured with a 2100 P Hatch Turbidimeter, while pH was measured with a WTW pH 315i meter. Secchi depth was measured with a 20 cm black and white Secchi disk. Water samples were collected by a Van Dorn sampler. Nutrient analysis was carried out following the methods outlined in [19]. Total nitrogen in the samples was analyzed on unfiltered samples by digestion with concentrated sulfuric acid (by autoclave procedure) to convert organic nitrogen to ammonium nitrogen, and then analysis for total nitrogen was carried out as outlined for ammonium nitrogen. Phosphate phosphorus was measured following the ascorbic acid method as outlined in [19].

Samples for determination of phytoplankton were collected from subsurface water. The water samples (25 mLs) were preserved in acidic Lugol's solution. Phytoplankton species identification and enumeration was done using inverted microscope at 400x magnification. Phytoplankton taxa were identified using the methods of [20]. Phytoplankton densities were estimated by counting all the individuals whether these organisms were single cells, colonies, or filaments. The resulting counts were used to calculate the algal density and expressed in cells/mL. The quantification of chlorophyll *a* was performed following the methods outlined in [21]. Chlorophyll *a* content of the water was determined in ug/L and algal densities in individuals, colonies, or filaments per litre.

### 2.3. Macrophyte Diversity Studies

Subjective and quantitative techniques were employed in the studies of aquatic vegetation or macrophytes. Transects were taken at different zones and percentage, cover was estimated with 1 × 1 m quadrats. Plants with diagnostic features such as flowers, fruits, shoots, and rhizomes were collected and correctly pressed and labeled with a brief habitat description and associated taxa. Macrophyte species occurring in the various sites were recorded, and the sites at which they occurred were marked by GPS. Identification of aquatic macrophytes was carried out by use of keys of [22–25]. Photographs were also taken using an HSC- 5 50 Sony model digital camera.

### 2.4. Invertebrates and Fish Associated with Macrophytes

Invertebrates were sampled with a 30 × 30 cm “kick-net” with a 0.5-mm mesh size by sweeping under the hyacinth mats or hippo grass. Snails were then separated from the collected root mass by vigorously shaking each root sample in a bucket containing 10% isopropyl alcohol, causing them to detach from the roots. Samples were sorted in a white plastic tray with clear water. The snails were identified in the field using taxonomic reference and taken to the laboratory for confirmatory identification. The assessment of the effect of the relative abundance of fish associated with the macrophytes was done by sampling using an electrofisher. Electrofishing activity was carried out using a Septa model unit which discharges voltages of up to 600 volts with accompanying Amperes of between 5 to 30 Amps. A pulsed mode of discharge was adopted for electrocution lasting 10 minutes at each attempt. Species identification followed descriptions given by [26]. 

## 3. Results and Discussion

### 3.1. Physicochemical Parameters

The physical and chemical characteristics of the sampled sites are given in Table 1. Results of the environmental parameters showed that dissolved oxygen ranged from 2.0 mgL^−1^ at Samunyi (Homa Bay) to 9.5 mgL^−1^ at Asembo Bay. Conductivity ranged from 156.6 *μ*Scm^−1^ at Lwanda Gembe to 176.0 *μ*Scm^−1^ at Samunyi (Homa Bay). The sampled sites from the different habitats had low transparency but elevated turbidity, total phosphorus (TP), total nitrogen (TN), chlorophyll *a*, and algal counts. The high nutrient concentrations provoked the proliferation of algal blooms in the hyacinth and hippo grass habitats. There was significantly higher turbidity in the hippo grass habitats compared to the water hyacinth and open water habitats (*P* = 0.009, one way ANOVA).

### 3.2. Macrophyte Inventory

Results of the inventory of macrophytes are shown in Table 2. An inventory of the macrophytes during the succession revealed that the dominant macrophytes were hippo grass *Vossia cuspidata* and water hyacinth *Eichhornia crassipes.* Other important aquatic plants identified were *Ipomoea aquatica, Enydra fluctuans, Cyperus papyrus *L., and *Aeschynomene elaphroxylon *(Guill. and Perr.) Taub. *Azolla pinnata *Decne ex Mett. and *Lemn*a sp. were only observed in areas where hippo grass had been cut while fishing for *Clarias gariepinus* juvenile, used as bait for Nile perch *Lates niloticus* fishery.

### 3.3. Macrophyte Succession

**Figure 2:**
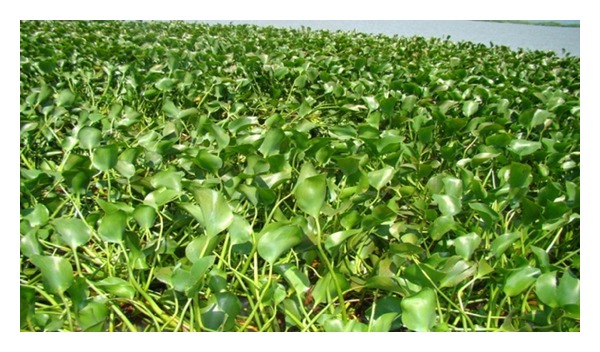
A pure population of the water hyacinth.

Several stages of macrophyte succession were noted during the study. These were then categorized as pure water hyacinth population, early, mid, late, and climax stages of succession. During pure water hyacinth stage, the plant community was entirely a population of water hyacinth (Figure 2). At the time of the study, such zones were rare and were only found at Homa Bay, Lwanda Gembe, and Asembo Bay on the shoreline in hotspots with high nutrient concentrations. Areas covered were also limited with each population cover rarely exceeding 500 m^−2^.

**Figure 3:**
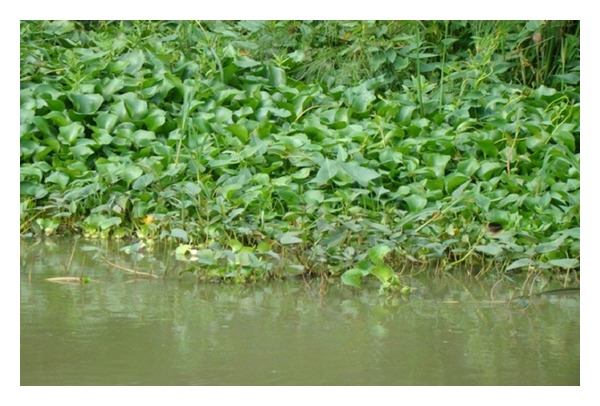
Water hyacinth mat showing encroachment by *Ipomoea aquatica* and *Enydra fluctuans*.

In the early stages of water hyacinth infestation, the weed takes foothold on the shoreline in the areas where native aquatic plants thrive. The early stages of water hyacinth succession occur in this zone and start when the pure water hyacinth mats were invaded by a plethora of opportunistic (usually emergent macrophytes) native invaders. The first common invaders were observed to be *Ipomoea aquatica *followed by *Enydra fluctuans* and an unidentified macrophyte of the Commelinaceae family. These are emergent runners which venture into the lake by creeping on the water hyacinth plants. In some areas, it is at this stage that scanty shoots of *Vossia cuspidata* started to appear among the other plants (Figure 3).

**Figure 4:**
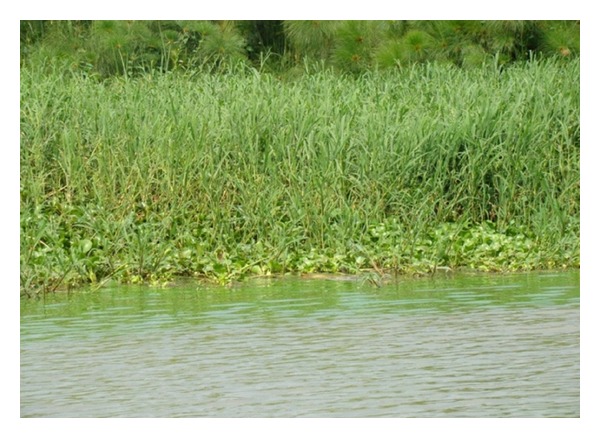
The late stages of macrophyte succession showing hippograss replacing the water hyacinth.

During the midstages of succession, the invader native aquatic plants were found to coexist within water hyacinth. It is at this stage that we observed hippo grass shoots within the macrophyte community consisting of the water hyacinth, *I. aquatica, E. fluctuans*, and a macrophyte of Commelinaceae family. With the increase of the opportunistic emergent invaders, there was an observed decrease in the proportion of the water hyacinth in the mat. During this stage, hippo grass had established itself within the community. Although the hippo grass is an emergent plant, its survival on water is by use of the water hyacinth as a substrate while proliferation within the community is because of the nutrients and detritus of the decaying water hyacinth. The buoyance of the hippo grass is provided by the water hyacinth biomass. The results of this succession are that the proportion of the water hyacinth decreases further in the mat. At the climax stage the water hyacinth is fully covered by the hippo grass owing to the fact that the hippo grass grows to height of 1.5 meters, while the water hyacinth grows to a height of 0.5 m (Figure 4).

The taller hippo grass shades the water hyacinth,* Ipomoea aquatic*,* and Enydra fluctuans* from sunlight. The *Ipomoea aquatica*, however, evades the shading effects of the hippo grass by climbing/twinning itself around the hippo grass. The shaded water hyacinth and *Enydra fluctuans *die off due to lack of sunlight contributing significantly to the organic matter (rich in nutrients) which fuels more proliferation of the hippo grass.

**Figure 5:**
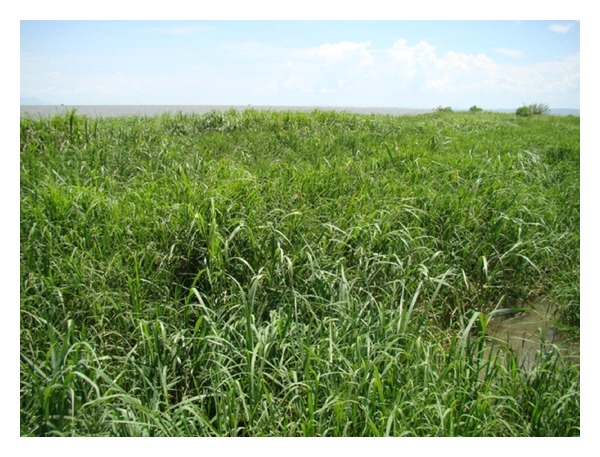
The climax stages of the macrophyte succession showing a single population of hippograss.

During heavy storms and wind activity, the population of the hippo grass is sloughed off the sheltered bays into the lake resulting into floating islands. The sloughing is aided by the compact mass of the hippo grass and its height (Figure 5).

**Figure 6:**
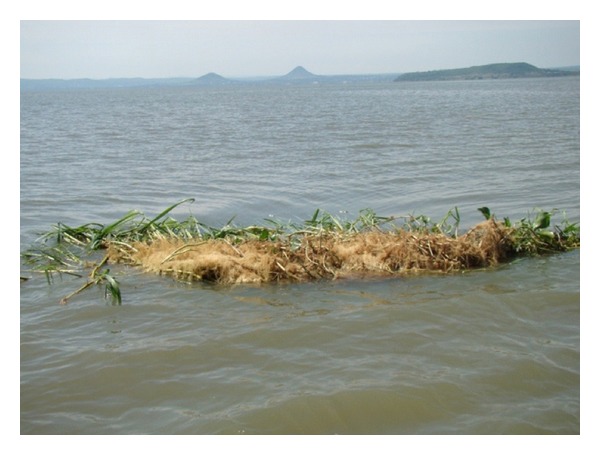
The sinking mats of hippograss after collapse of the water hyacinth substrate.

After the nutrient-rich heterotrophic layer substrate of the dying water hyacinth is exhausted, the hippo grass (now existing as floating islands in the lake) starts to die off since it cannot extract nutrients from the water as it is an emergent plant living on the shoreline extracting the nutrients from the substrate in the littoral zones of the lake (Figure 6).

**Figure 7:**
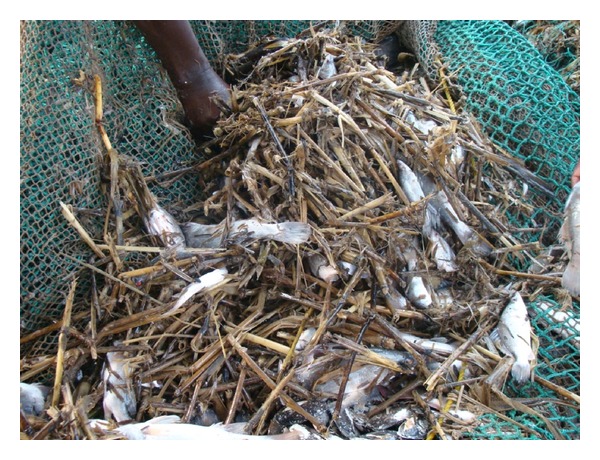
Fragments of the hippograss at the bottom of the lake as obtained from the bottom trawls of the sampled sites.

The few mats of water hyacinth existing under the mat do not sink with the mat but float out into the open water to start the new colonies of the water hyacinth mats. Observations from trawl surveys indicated that large fragments of fleshly sunk hippo grass had sunk at the bottom of the lake. Initial estimates revealed that more than 3,000 hectares of hippo grass could have sunk to the bottom of the lake (Figure 7).

**Figure 8:**
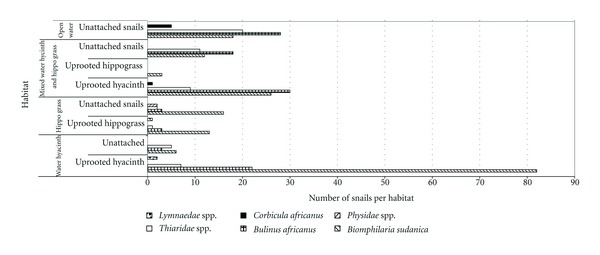
Relative abundance of different invertebrate species collected in the habitats of water hyacinth and hippo grass and in open water at different sites within the Nyanza Gulf of Lake Victoria.

Results on the distribution of invertebrates are shown in Figure 8. The study found a strong association of snails associated with aquatic macrophytes including *Biomphalaria sudanica *and *Bulinus africanus,* the two most common hosts for schistosomiasis in the Nyanza Gulf of Lake Victoria.

**Figure 9:**
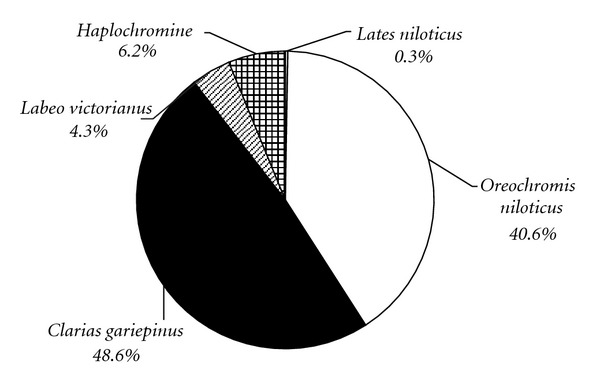
% composition of the fish species in the areas covered by water hyacinth complex.

The inventory and abundance composition of fish species from areas covered by water hyacinth, hippo grass, or both varied from that of the open water obtained using trawls. In this habitat, *Clarias gariepinus* populations dominated, contributing up to 48.6% of the biomass. *Oreochromis niloticus* is the second most abundant species, contributing 40.6% of the biomass (Figure 9). An endemic species like *Labeo victorianus *was also found under the mats with a composition of 4.3% of the total catch.

**Figure 10:**
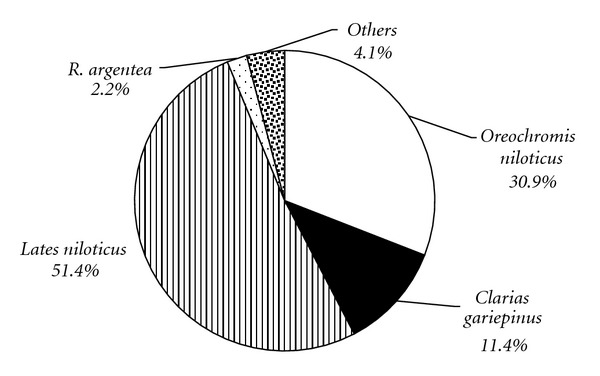
Percent (%) composition of the fish species in the open water areas.

The most abundant species in the open lake was *Lates niloticus* which accounted for 51.4% of the biomass. *O. niloticus *contributed 30.9% of the total trawl biomass (Figure 10). *Clarias gariepinus* is the 3rd most abundant fish species except for *R. argentea* and C*aridina niloticus *(both captured in the codend 10 mm mesh). From the aforementioned, it is apparent that macrophyte succession has the capacity to alter aquatic biodiversity in the lake. The most important factor contributing to these changes was dissolved oxygen.

The main anoxic tolerant species are *C. gariepinus*, *O. niloticus, Synodontis *spp., and *P. aethiopicus*, while *Lates niloticus *prefers areas with high oxygen levels.

Before 1970s, the shoreline wetlands of Lake Victoria were dominated by the emergent *C. papyrus*. During this period, establishment of submerged plants in the inshore areas was hindered by the constant disturbance of the substrate by the once dominant haplochromine fish species. For instance, during the peak biomass of haplochromines in Lake Victoria, before 1960s, the fishes hindered the establishment of macrophytes in the inshore areas through enhancement of turbidity by disturbing the bottom substrate and sediments [27]. These papyrus dominated mats frequently sloughed off river mouths during the rainy seasons and formed floating islands in the offshore areas. *Vossia cuspidata* was mainly found at the banks of potamon sections of major affluent rivers. Before the invasion of water hyacinth in the 1990s, the only other free-floating macrophytes in the lake were *Pistia stratiotes, Azolla pinnata, A. nilotica*, and *Lemna* sp. The common floating leafed species included *Trapa natans *and* Nymphaea lotus* while the submerged were *Potamogeton schweinfurthii*, *Vallisneria spiralis *L., *Ceratophyllum demersum *L., and *Najas horrida*. According to [28] the aquatic macrophyte, communities of the wetlands are a part of a vegetation continuum from land to below water. Frequently, though there are distinct zones of vegetation along the continuum. In this study, approaching the wetland from dry land, the dominant macrophytes were found to consist of *Phragmites australis* and stands of *Typha domingensis*. Immediately after this, the *Cyperus papyrus* was the most dominant consisting mainly of mono specific stands intertwined with creepers such as *Vigna lutea *(Del.) Hook. and *Ipomoea aquatica var. aquatica.* Next in line from the papyrus zone was a thin strip of hippograss *Vossia cupsidata* interspersed with *Echinochloa pyramidalis.* The lake-swamp interface zone was colonized by the floating mats of *Eichhornia crassipes*. On establishment, the massive mats of the hyacinth overwhelmed these other macrophytes by competing for nutrients, smothering, and cutting out sunlight. The impact of water hyacinth could have led to the extinction of *Azolla nilotica* which was common at the mouth of River Nyando [11, 29]. Results from this study showed that water hyacinth is the genesis of plant succession phenomenon in the Nyanza Gulf of Lake Victoria. Water hyacinth established itself in the Nyanza Gulf after the short rains of 2006 (Kusewa pers. comm). The main control of water hyacinth is through biological method using *Neochetina eichhorniae* and *Neochetina bruchi.* The dying plants of the water hyacinth provide a substrate for emergent macrophytes, and this results in rapid increase of these especially *Vossia cuspidata, Ipomoea, aquatica, Enydra fluctuans*, and unidentified macrophyte of the family Commelinaceae. Our observation revealed that the macrophytes that initiate the colonization on the water are *Enydra fluctuans* and *Ipomoea aquatica* these plants are closely followed by the hippograss. Initially, the hippograss seems to coexist with the water hyacinth in a mutually beneficial association such that both plants extend their range along the shore and out into the open water. The mats that develop are mainly dominated by opportunistic native plants such as *Vossia cuspidata, Ipomoea aquatica var. aquatica, Enhydra fluctuans*, and a macrophyte of the Commelinaceae family. The flourishment of the floating islands is nourished by the nutrients from the water hyacinth substrate, and the cohesion is provided by the underwater biomass of the water hyacinth. According to [30], freshwater hyacinth plant contains high levels of nutrients. These nutrients enhance the proliferation of the hippo grass and its associated vegetation. According to [17], light is an important limiting factor to water hyacinth growth. Light becomes nonlimiting to CO_2_ uptake at a photosynthetic active radiation (PAR) of 2000 *μ*E m^−2^ s^−1^. In Lake Victoria, this light level occurs for about 6 h around midday. [15, 17], associated the decline of the water hyacinth in 1998 in Lake Victoria to climatic perturbations leading to the decreased 9 light intensity occasioned by the cloudy, wet *El Nino *weather phenomenon of 1997-1998 [31]. Prolonged suboptimal light will reduce growth and reproduction rates and relatively increase the effect of other debilitating influences such as other weather-related factors, for example, water level, wave action, water quality, temperature, and humidity as well as weevil herbivory and phytopathogenic attack [17, 18, 32]. In this study, we observed that the reduced light conditions were majorly caused by the shading effects of the water hyacinth by the hippo grass. Whereas the water hyacinth is a perennial aquatic herb; rhizome and stems normally floating, rooting at the nodes, with long black pendant roots, it only grows generally to a height of 0.5 m [33]. The hippo grass is perennial with submerged or floating culms. It has a two to six on a short axis, or solitary 15–22 cm long; sessile spikelets up to 10 mm long, lower glume of both spikelets with a winged tail 5–30 mm long, rarely shorter. The plant can grow to a total height of 1.5 metres. The taller hippo grass shades the water hyacinth,* Ipomoea aquatica, Enydra fluctuans*, and a macrophyte of the Commelinaceae family from sunlight, heat, and UV radiation. The *Ipomoea aquatica*, however, evades the shading effects of the hippo grass by climbing/twinning itself around the hippo grass. The shaded water hyacinth and *Enydra fluctuans *die off due to lack of sunlight contributing significantly to the organic matter (rich in nutrients) which fuels the more proliferation of the hippo grass. During heavy storms, strong wind activity, or currents, the population of the hippo grass is sloughed off the sheltered bays into the lake resulting into floating islands. The sloughing is aided by the compact mass of the hippo grass and its height. The fate of the floating islands is determined by the time; it takes for total disintegration of the heterotrophic substrate. After the nutrient-rich heterotrophic layer substrate of the dying water hyacinth is exhausted, the hippo grass (now existing as floating islands in the lake) starts to die and sink to the bottom of the lake.

## 4. Conclusion

This study provides compelling evidence to show that macrophyte succession has the capacity to alter aquatic biodiversity in the lake. For instance, areas dominated by water hyacinth and hippo grass were found to be anoxic and dominated by anoxic tolerant species such as *C. gariepinus*, *O. niloticus, Synodontis *spp., and *P. aethiopicus.* Areas without water hyacinth were dominated by *Lates niloticus. *In addition, the succession threatened endemic macrophytes such as the free-floating *Pistia stratiotes *and *Azolla pinnata.*

Water hyacinth infestation is a symptom of broader watershed management and pollution problems in the Nyanza Gulf of Lake Victoria. Methods for water hyacinth control should include reduction of nutrients in the water bodies. This can be achieved through treatment of waters flowing from sewage works and factories at least up to tertiary level using constructed wetlands and reducing the diffuse loadings by changing land use practices as well as development of holistic management of pollution involving land, water, and air shed. There is need to implement an integrated approach to water hyacinth management in which biological control agents play the central role with leverages on manual/mechanical controls in a multifaceted approach. Research indicates that the present control programmes are expensive and do not provide any returns on investments. Most are self-propagating and return nutrient back into the system. The paradigm shift is emphasized for water hyacinth control through harvesting and utilization which is eco-friendly. Communities have expressed willingness to participate in community-based water hyacinth control strategies. The most practical approach is to involve them in manual and biological control activities, for example, in rearing weevils. Some little incentives may be necessary to leverage for lost time. The most sustainable method however includes organizing communities into cottage industrial production units with water hyacinth as raw material, and this is value added because an economic use is found for an unwanted plant. In this way, water hyacinth can provide a substitute for bulking agents, reducing the requirements for expensive goods, and generating income (through creation of employment, generating income) and improve standards of living from sale of byproducts providing alternative livelihoods and relieving the pressure from the fishery, forestry, and energy.

## Figures and Tables

**Table 1:** Variations (Mean ± SD) in the physicochemical parameters between the habitats of water hyacinth complex. Values in parenthesis indicate the range.

Habitat parameter	Water hyacinth	Hippo grass	Mixed (water hyacinth and hippo grass)	Open water
Secchi depth (m)	0.35 ± 0.13	0.23 ± 0.10	0.31 ± 0.14	0.42 ± 0.12
	(0.10–0.45)	(0.15–0.35)	0.15–0.45	(0.25–0.60)
Temp (°C)	28.50 ± 2.39	26.80 ± 2.33	28.00 ± 1.77	27.70 ± 2.10
	(25.70–31.90)	(24.20–28.70)	(25.90–30.20)	(25.50–31.8)
pH	7.80 ± 0.82	7.40 ± 1.36	7.60 ± 0.40	7.80 ± 0.47
	(6.60–8.80)	(5.90–8.50)	(7.20–8.10)	(7.20–8.50)
Turbidity (NTU)	112.20 ± 111.79	461.30 ± 360.13	152.80 ± 121.89	71.1 ± 24.51
	(54.00–339.00)	(77.00–791.00)	(78.30–335.00)	(47.30–115.00)
TP (*μ*gP/L)	538.70 ± 823.37	486.9 ± 444.77	391.90 ± 147.04	208.10 ± 47.78
	(146.90–2216.90)	(179.70–996.90)	(179.70–511.10)	(149.70–284.00)
TN (*μ*gN/L)	975.20 ± 796.49	542.4 ± 139.30	587.80 ± 157.18	633.30 ± 118.73
	(750.40–2584.20)	(517.30–692.50)	(396.30–726.70)	(485.70–834.60)
Chloro-*a *(*μ*g/L)	9.90–5989.40	231.90 ± 367.83	301.80 ± 245.82	89.95 ± 76.81
	(1045.90 ± 1045.00)	(19.40–656.70)	(11.20–612.30)	(9.90–200.20)
Algal densities (individuals/cells/colonies/L)	12172.5 ± 16177.38)	6914.67 ± 9340.42	8058.25 ± 5731.59	4772.12 ± 4264.31
	(700.00–36120.00)	(1493.00–17700.00)	(1652.00–15522.00)	(1116.00–13638.00)

**Table 2:** An inventory of the macrophyte vegetation and the proportions (%) observed at the sampled sites.

Station	Vegetation	Proportion (%)
Dunga beach	Hippo grass *Vossia cuspidata *(Roxb.) Griff.	98
	Water hyacinth* Eichhornia crassipes *(Mart.) Solms-Laubach	1.5
	Nile cabbage/water lettuce *Pistia stratiotes *L.	0.5

Kibos	Water hyacinth* Eichhornia crassipes *	50
	Hippo grass *Vossia cuspidata *	50

Sondu Miriu at interface zone	Water hyacinth* Eichhornia crassipes *	50
	Hippo grass,* Vossia cuspidata *	50

Homa Bay at Samunyi—A	Water hyacinth*, Eichhornia crassipes *	90
	Swamp cabbage/water spinach *Ipomoea aquatica var. aquatica* Forsk.	8
	*Enydra fluctuans *Lour.	1.5
	Hippo grass *Vossia cuspidata *	0.5

Homa Bay at Samunyi—B	Water hyacinth* Eichhornia crassipes *	90
	Swamp cabbage/water spinach *Ipomoea aquatica var. aquatica *	2
	*Enydra fluctuans* Lour.	8

Homa Bay at Samunyi—C	Water hyacinth *Eichhornia crassipes *	60
	Hippo grass *Vossia cuspidata *	30
	*Enydra fluctuans *Lour.	8
	Swamp cabbage/water spinach *Ipomoea aquatica var. aquatica *	2

Homa Bay (Floating mat)	Hippo grass *Vossia cuspidata *	85
	Water hyacinth* Eichhornia crassipes *	10
	*Enydra fluctuans*	2
	Swamp cabbage/water spinach *Ipomoea aquatica var. aquatica *	1
	Common papyrus *Cyperus papyrus *L.	1
	Ambach tree* Aeschyonomene elaphroxylon *(Guill. and Perr.) Taub.	1

Homa Bay offshore (floating mat)	Water hyacinth* Eichhornia crassipes *	99
	Swamp cabbage/water spinach *Ipomoea aquatica var. aquatica *	1

Oluch river mouth	Hippo grass *Vossia cuspidata *	99
	Water hyacinth* Eichhornia crassipes *	1

Lwanda Gembe—A	Water hyacinth* Eichhornia crassipes Ipomoea aquatica var. aquatic *	95
	Swamp cabbage/water spinach	1
	Commelinaceae	2
	*Enydra fluctuans*	2

Lwanda Gembe—B	Unidentified macrophyte—B	90
	Water hyacinth* Eichhornia crassipes *	5
	*Polygonum setosulum *Meisn.	5

Lwanda Gembe—C	Water hyacinth* Eichhornia crassipes *	80
	*Enydra fluctuans*	15
	Unidentified macrophyte—B	4
	*Polygonum setosulum*	1

Lwanda Gembe—D	Water hyacinth* Eichhornia crassipes *	90
	Hippo grass *Vossia cuspidata *	5
	*Enydra fluctuans*	5

Asembo Bay—A	Hippo grass *Vossia cuspidata *	100

Asembo Bay—B	Hippo grass *Vossia cuspidata *	80
	Swamp cabbage/water spinach *Ipomoea aquatica var. aquatica *	20

Asembo Bay—C	Hippo grass *Vossia cuspidata *	80
	Water hyacinth* Eichhornia crassipes *	20
Asembo Bay—D	Hippo grass *Vossia cuspidata *	90
	Water hyacinth* Eichhornia crassipes *	9
	Swamp cabbage/water spinach *Ipomoea aquatica var. aquatica *	1

Asembo Bay—E	Water hyacinth* Eichhornia crassipes *	50
	Hippo grass *Vossia cuspidata *	20
	Water fern, Water velvet* Azolla pinnata *	15
	*Enydra fluctuans *	14
	Duckweed* Lemna *sp.	1

Asembo Bay	Water hyacinth* Eichhornia crassipes *	90
	Hippo grass *Vossia cuspidata *	10
